# A circulating miR-19b-based model in diagnosis of human breast cancer

**DOI:** 10.3389/fmolb.2022.980841

**Published:** 2022-09-16

**Authors:** Qian Zhao, Lei Shen, Jinhui Lü, Heying Xie, Danni Li, Yuanyuan Shang, Liqun Huang, Lingyu Meng, Xuefeng An, Jieru Zhou, Jing Han, Zuoren Yu

**Affiliations:** ^1^ Research Center for Translational Medicine, Shanghai East Hospital, Tongji University School of Medicine, Shanghai, China; ^2^ Department of Surgery, Shanghai East Hospital, Tongji University School of Medicine, Shanghai, China; ^3^ Jinzhou Medical University, School of Basic Medical Sciences, Jinzhou, Liaoning, China; ^4^ Department of Physical Examination, Shanghai East Hospital, Tongji University School of Medicine, Shanghai, China

**Keywords:** breast cancer, circulating miRNA, biomarker, diagnosis model, miR-19

## Abstract

**Abstract Objective:** Breast cancer (BC) is becoming the leading cause of cancer-related death in women all over the word. Identification of diagnostic biomarkers for early detection of BC is one of the most effective ways to reduce the mortality.

**Methods:** Plasma samples from BC patients (*n* = 120) and normal controls (*n* = 50) were collected to determine the differentially expressed circulating miRNAs in BC patients. Binary logistic regression was applied to develop miRNA diagnostic models. Receiver operating characteristic (ROC) curves were applied to calculate the area under the curve (AUC). MMTV-PYMT mammary tumor mice were used to validate the expression change of those circulating miRNAs. Plasma samples from patients with other tumor types were collected to determine the specificity of the model in diagnosis of BC.

**Results:** In the screening phase, 5 circulating miRNAs (miR-16, miR-17, miR-19b, miR-27a, and miR-106a) were identified as the most significantly upregulated miRNAs in plasma of BC patients. In consistence, the 5 miRNAs showed upregulation in the circulation of additional 80 BC patients in a tumor stage-dependent manner. Application of a tumor-burden mice model further confirmed upregulation of the 5 miRNAs in circulation. Based on these data, five models with diagnostic potential of BC were developed. Among the 5 miRNAs, miR-19b ranked at the top position with the highest specificity and the biggest contribution. In combination with miR-16 and miR-106a, a miR-19b-based 3-circulating miRNA model was selected as the best for further validation. Taken the samples together, the model showed 92% of sensitivity and 90% of specificity in diagnosis of BC. In addition, three other tumor types including prostate cancer, thyroid cancer and colorectal cancer further verified the specificity of the BC diagnostic model. **Conclusion:** The current study developed a miR-19b-based 3-miRNA model holding potential for diagnosis of BC using blood samples.

## Introduction

Breast cancer is the most frequent malignancy in women worldwide. Since 2020, female breast cancer has become the most commonly diagnosed cancer (Sung et al., 2021). Although 70%–80% of breast cancer patients at early and non-metastatic stages have good prognosis after surgery, it remains incurable after metastasis (Harbeck et al., 2019). As such, early diagnosis is considered as an important way leading to personalized immediate treatment, longer survival and better prognosis in breast cancer (Wulfkuhle et al., 2003).

The current approaches for diagnosis of breast cancer mainly rely on imaging examinations including ultrasound, ammography and magnetic resonance examination (MRI). Although liquid biopsy tests have been frequently applied in early cancer diagnosis by analyzing DNA, RNA, proteins, or exosomes in bodily fluids (Alimirzaie et al., 2019), it is still unavailable for breast cancer due to lack of a reliable and specific biomarker. There is an urgent need to develop more effective approaches for detection of breast cancer.

microRNAs (miRNAs) are a class of small non-coding RNA playing crucial roles in occurrence and development of human diseases including cancer. Approximately 50% of annotated miRNAs were located at fragile sites related to cancer (Bevacqua et al., 2021). Aberrant expression of miRNAs has been frequently reported in various types of tumors (Rao et al., 2012; Su et al., 2012; Mulrane et al., 2013; Yoshino et al., 2013). Some miRNAs show tissue-specific or tumor type-specific manner in expression (Ludwig et al., 2016; Deng et al., 2019; Chen et al., 2020). Furthermore, miRNAs can be secreted into body fluids, including blood, urine, cerebrospinal fluid, or saliva, which are called circulating miRNAs (Mitchell et al., 2008; Kosaka et al., 2010). It has been widely demonstrated that circulating miRNAs have potential to be used as diagnostic and/or prognostic biomarkers or playing other functions in cancer (Mitchell et al., 2008; Ding et al., 2020). For example, circulating miR-1268b and miR-6075 in serum were identified as diagnostic markers of lung cancer (Asakura et al., 2020). This two-miRNA-based model displayed high sensitivity regardless of histological type and pathological TNM stage of the cancer (Asakura et al., 2020). miR-21 and miR-1,246 were enriched in human breast cancer exosomes, and significantly elevated in the plasma of patients with breast cancer (Hannafon et al., 2016). Combination of miR-1,246 and miR-21 in plasma showed a better indicator of breast cancer than either of them (Hannafon et al., 2016).

In the current study, we performed a miRNA screening analysis using plasma samples from breast cancer patients. A subset of circulating miRNAs was identified having aberrant expression in association with breast cancer, such as miR-16, miR-19b, miR-106a, miR-17, and miR-27a. By combining miR-19b, miR-16, and miR-106a, a 3-circulating miRNA model was developed for breast cancer diagnosis. As high as 92% of sensitivity and 90% of specificity were obtained. Our findings suggest a novel approach to analyze the circulating miRNAs as indicative of the occurrence of breast cancer. A larger cohort of patients with BC will be necessary for further validation.

## Materials and methods

### Patient cohorts

A total of 234 plasma samples were collected from Shanghai East Hospital and Shanghai Huangpu Hospital, including 120 BC samples, 16 prostate cancer samples, 15 thyroid cancer samples, 33 colorectal cancer samples and 50 age-matched female controls. 40 BC patients and 20 normal controls were applied in the screening cohort, and additional 80 BC patients and 30 normal controls for validation and model development. 16 samples with prostate cancer, 15 with thyroid cancer and 33 with colorectal cancer were used for the external specificity validation. Clinical characteristics of the patients were listed in Supplementary Table S1–S3. The study was approved by the Institutional Review Board (IRB) of Shanghai East Hospital, Tongji University School of Medicine. All subjects were provided with a written informed consent.

### Animals

Animal studies were approved by the Institutional Animal Care and Use Committee of the Tongji University School of Medicine. MMTV-PYMT FVB mice were presented by Professor Suling Liu at Fudan University. All experiments were performed in accordance with the relevant guidelines and regulations for animal use.

### Plasma collection and RNA extraction

Five mL (patients) or 1 mL (mice) blood samples were collected using EDTA-treated blood collection tubes. Plasma was isolated by centrifugation at 2,000 rpm for 5 min at 4 °C, and stored 200 μL aliquots in -80 °C freezer for further analysis. Total RNA was extracted using Trizol reagent (Invitrogen, United States) according to standard procedure. Glycogen was used as an inert carrier to make RNA pellet visible. RNA quality was analyzed using Agilent Bioanalyzer 2,100.

### miRNA QRT-PCR analysis

100–200 ng of total plasma RNA was applied to prepare the first strand cDNA of miRNAs by using the M&G miRNA Reverse Transcription kit (miRGenes, China) following the manufacturer’s instruction. The SYBR Green Master Mix (Applied Biosystem, United States) and QuantStudio™ 6 Flex Real-Time PCR System (Applied Biosystem, United Ststes) were used for real-time PCR analysis. 5 s rRNA was used for normalization. Forward primer sequences of all miRNAs were presented in Supplementary Table S1. All primer oligos were synthesized by GenScript (Nanjing, China).

### Diagnostic model development

The miRNA diagnostic model was developed by binary logistic regression using IBM SPSS Statistics 26 software. Based on the relevant parameters of binary logistic regression, the relationship between dependent Y scores (Control and BC as dependent variables) and independent values (miR-16, miR-19b, miR-106a, miR-17, and miR-27a as independent variables) was analyzed. Y score of 0.5 was set for cutoff.

### Databases

The TCGA pan-cancer database (https://www.xiantao.love/products) and a Kaplan-Meier plotter tool were used to analyze the gene expression levels and overall survival.

### Statistical analysis

Two-tailed *t*-test was used to analyze the independent samples. For statistical analysis of miRNA expression, log transformation was performed on the values (2^^-∆∆Ct^) to obtain the normal distribution of miRNA expression levels (Kuwabara et al., 2011; Yao et al., 2014). MeV 4.9 software was used to generate the heatmap. *p* < 0.05 was considered as statistically significant difference. GraphPad Prism V8.0 software was used for figure preparation.

## Results

### Characteristics of subjects

**FIGURE 1:**
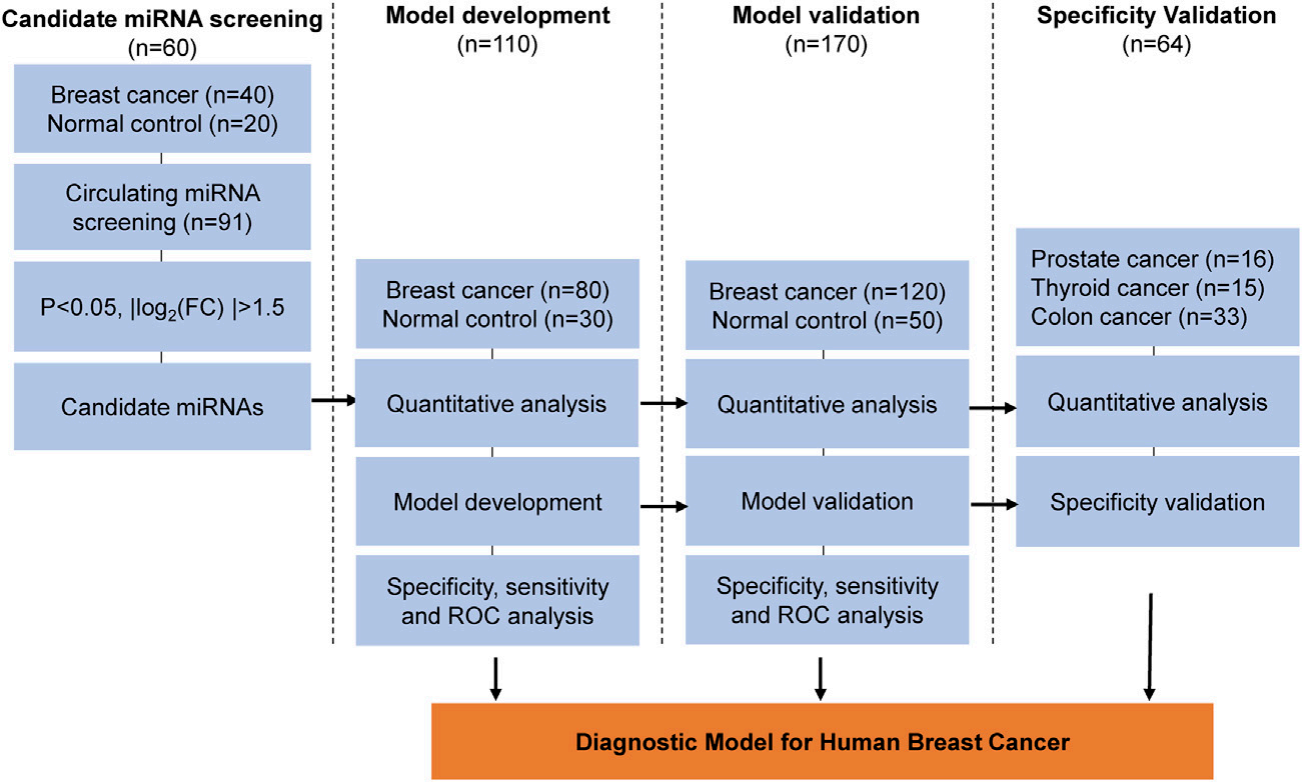
Overview of the study design.

A total of 234 subjects, including 120 with breast cancer, 16 with prostate cancer, 15 with thyroid cancer, 33 with colorectal cancer, and age-matched 50 normal controls were included in the current study. All the characteristics of the subjects were shown in Supplementary Table S2, S3. The flow chart of the study design was shown in Figure 1.

### Identification of the circulating miRNA signature in the plasma of breast cancer

**FIGURE 2:**
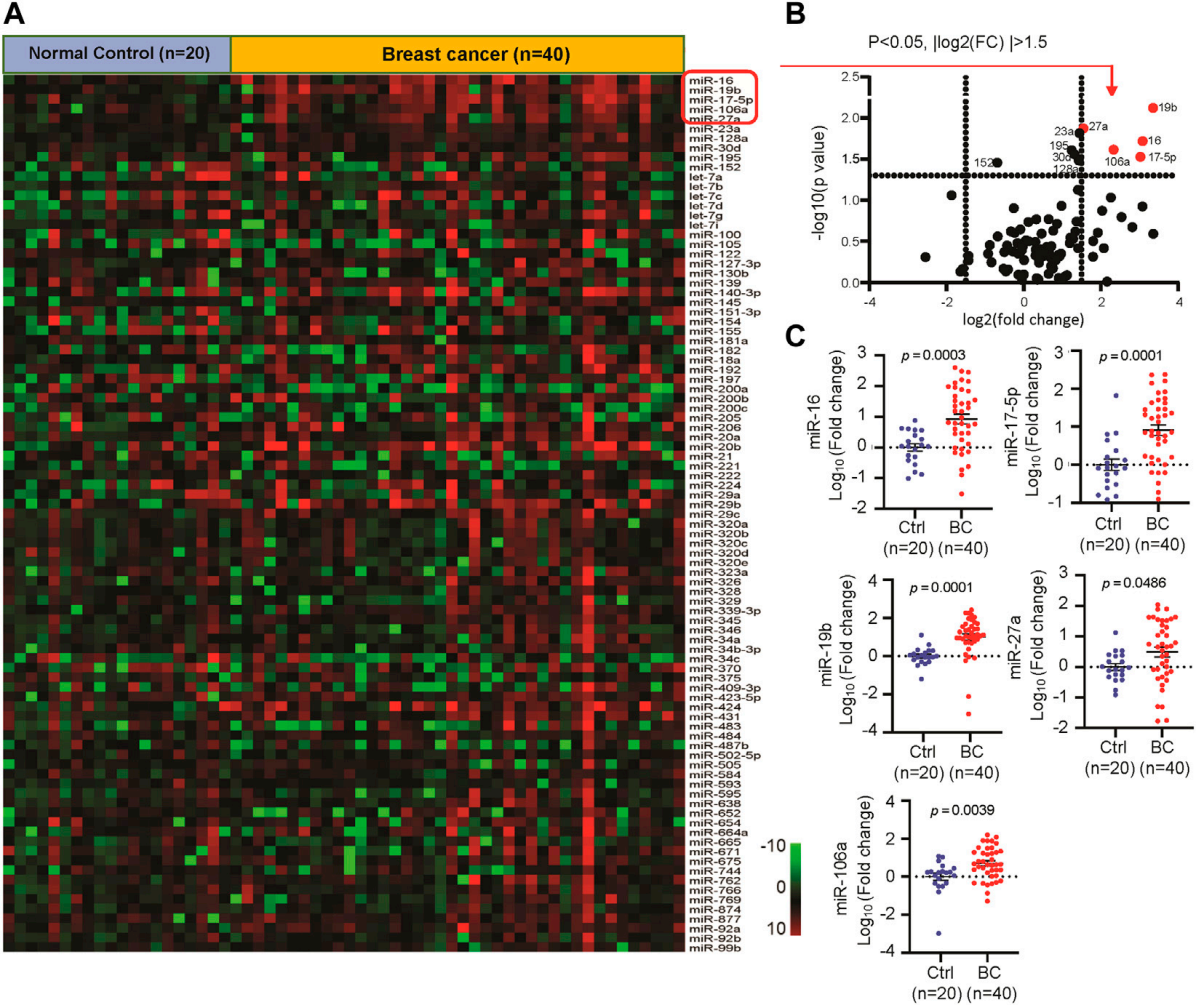
Identification of the differentially expressed circulating miRNAs in breast cancer (BC) patients. **(A)**. Heatmap of a quantitative RT-PCR based screening analysis of circulating miRNAs in the plasma of BC patients (*n* = 40) and normal controls (*n* = 20). **(B)**. Volcano plot reporting *p* values against fold changes, in which -log 10 (*p*-value) for miRNAs (*Y*-axis) plotted against their respective log 2 (fold change) (*X*-axis). 10 miRNAs with significant change (*p* < 0.05) were labeled with ID. The red dots represented top five up-regulated miRNAs in BC. **(C)**. The relative expression levels of circulating miR-16, miR-17, miR-27a, miR-19b and miR-106a in 40 BC and 20 control samples.

**FIGURE 3:**
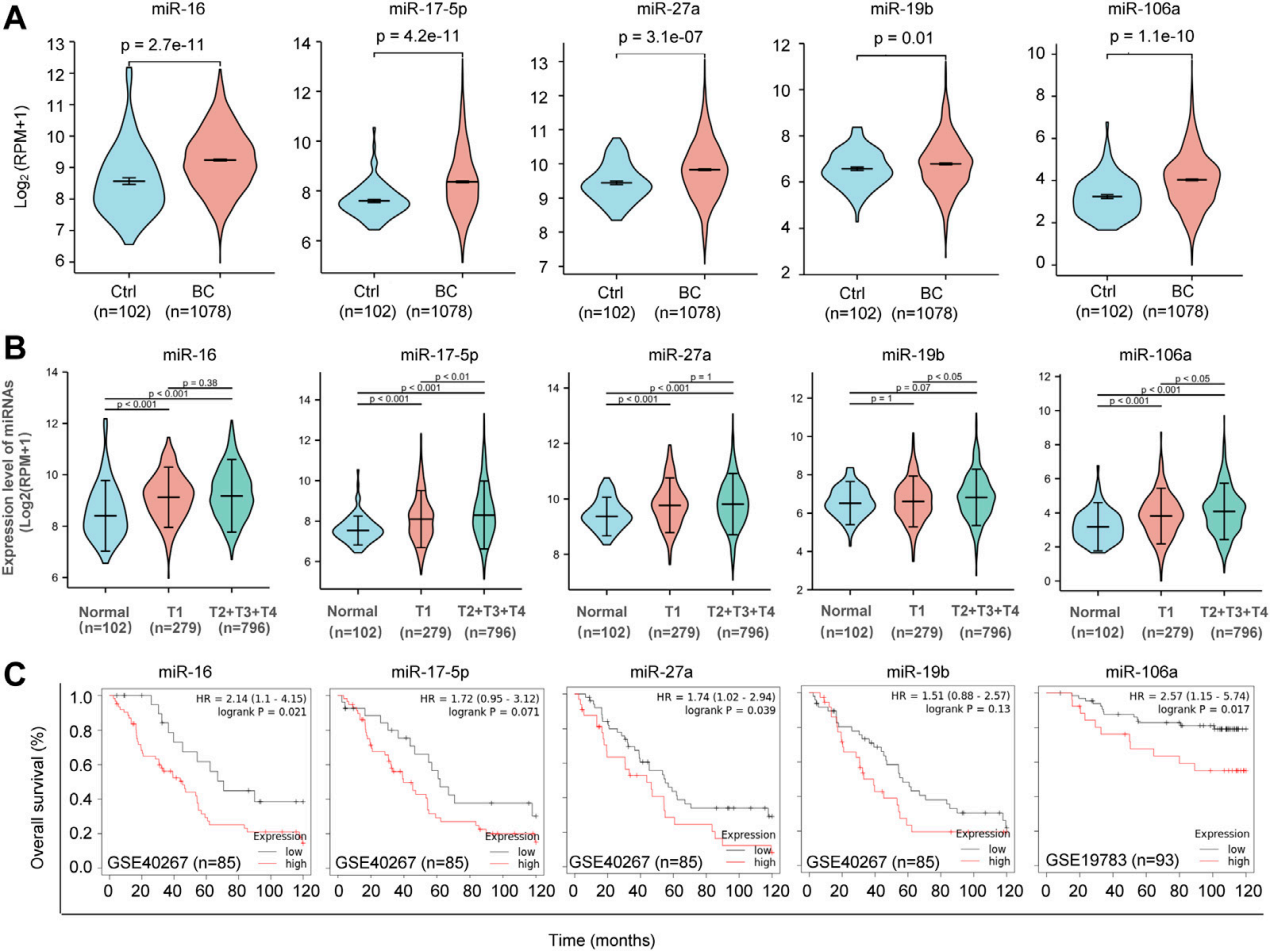
Clinical relevance of the five candidate miRNAs. **(A)**. Violin Plots showing significant upregulation of miR-16, miR-17, miR-27a, miR-19b and miR-106a in 1,078 BC patients and 102 normal controls. TCGA database was applied for analysis. **(B)**. Tumor stage-based analysis of miR-16, miR-17, miR-27a, miR-19b and miR-106a in 102 normal controls, 279 BC patients at tumor stage T1 and 796 BC patients at tumor stage later than T1. **(C)** Kaplan-Meier survival curves showing negative correlations between the overall survival rate and the expression levels of miR-16, miR-17, miR-27a, miR-19b and miR-106a in BC, respectively.

In order to identify the circulating miRNAs with differential abundance in the plasma of BC patients, plasma samples collected from 40 BC patients and 20 normal controls were applied for miRNA screening analysis. A RT-PCR-based home-made miRNA panel containing 91 tumor-related human miRNAs (Supplementary Table S4) was used. As a result, we identified 10 miRNAs with significant change (*p* < 0.05) in abundance (Figures 2A,B), in which miR-16, miR-19b, miR-106a, miR-17, miR-27a, miR-23a, miR-30d, miR-128a, and miR-195 showed significant upregulation, while the rest of miR-152 showed downregulation in the plasma of BC patients. After cutoff with (|log2(FC)|) >1.5 and *p*-value <0.05, miR-16, miR-19b, miR-106a, miR-17, and miR-27a were screened out as the best candidates of our interest (Figure 2B). The expression patterns of the five candidate miRNAs indicated the higher levels in the plasma of BC patients than that in normal controls (Figure 2C). Additional analysis of the 5 miRNAs in 1,078 BC samples and 102 normal controls from TCGA database further confirmed the upregulation of miR-16, miR-19b, miR-106a, miR-17, and miR-27a in BC (Figure 3A) in a tumor stage-dependent manner (Figure 3B) and a subtype-dependent manner (Supplementary Figure S1). They showed increase in patients at early-stage *vs.* normal controls and in patients at late stages *vs.* early stage (Figure 3B). And higher levels of the 5 miRNAs were observed in basal-like BC patients, compared to luminal subtype (Supplementary Figure S1). In addition, negative correlations between the expression levels of the five miRNAs and the overall survival were indicated, respectively (Figure 3C).

### Diagnostic model development with the circulating miRNA signature

**FIGURE 4:**
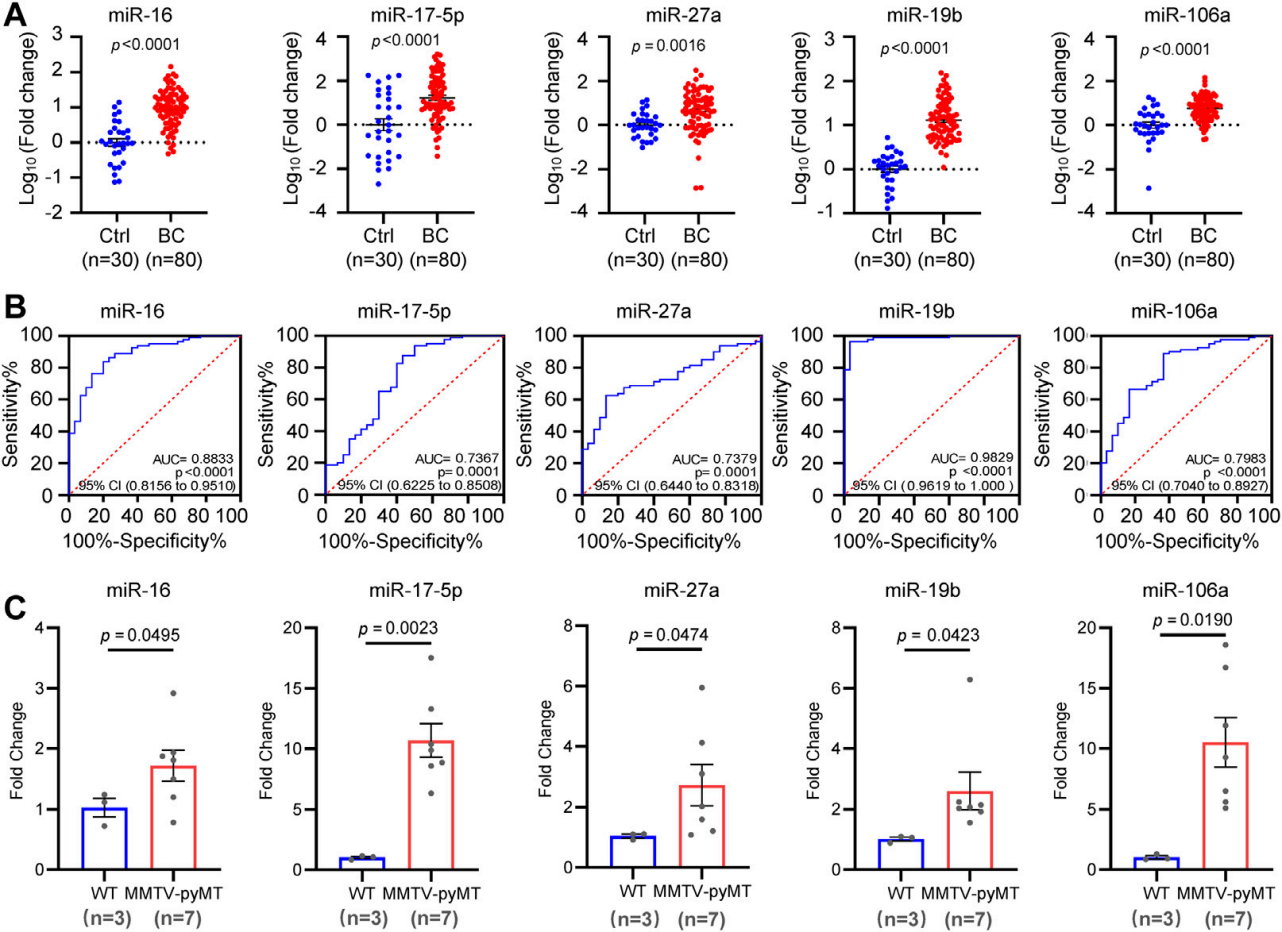
Independent validation of the five candidate miRNAs. **(A)**. Expression validation of circulating miR-16, miR-17, miR-27a, miR-19b and miR-106a in an independent cohort including 80 BC patients and 30 controls by quantitative RT-PCR analysis. **(B)**. Receiver operating characteristic (ROC) curves of miR-16, miR-17, miR-27a, miR-19b and miR-106a in the validation cohort. The values of AUC, 95% CI and *p* were indicated in the graphs. **(C)**. Upregulation of miR-16, miR-17, miR-27a, miR-19b, and miR-106a in plasma samples from MMTV-PYMT mammary tumor mice (*n* = 7) than that in normal controls (*n* = 3).

In order to determine the diagnostic potential of the circulating miR-16, miR-19b, miR-106a, miR-17, and miR-27a, additional 80 BC plasma samples and 30 normal controls were used to validate the expression change of the 5 miRNAs. As shown in Figure 4A, circulating miR-16, miR-19b, miR-106a, miR-17, and miR-27a showed higher level in BC patients than that in normal controls. Receiver operating characteristic (ROC) curve was introduced to assess the diagnostic potential of each miRNA. The area under curve (AUC) for miR-16, miR-17, miR-27a, miR-19b, and miR-106a was 0.8833, 0.7367, 0.7379, 0.9829, and 0.7983, respectively (Figure 4B). In order to further confirm the expression changes of these miRNAs, a MMTV-PYMT transgenic mice model was applied to develop mammary tumors. Analyses of miR-16, miR-17, miR-27a, miR-19b, and miR-106a demonstrated significant upregulation in plasma of mammary tumor mice, compared to normal control mice (Figure 4C).

Based on the expression information of the five miRNAs, binary logistic regression method was applied to develop diagnostic models. As a result, five models covering different miRNA combinations were derived, in which Y score >0.5 was considered as BC. The AUC value, diagnostic sensitivity and diagnostic specificity for each model were obtained by the ROC analysis (Supplementary Figure S2). The results suggested that miR-19b had the biggest contribution to the diagnostic models than other 4 miRNAs, and combination of multiple miRNAs had higher diagnostic accuracy.

### Accuracy and specificity validation of the model

**FIGURE 5:**
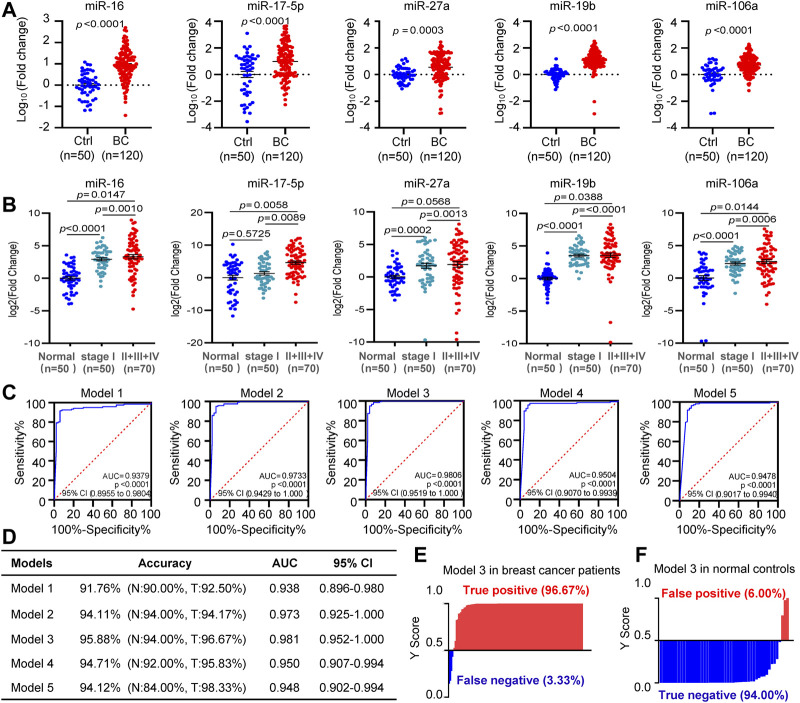
Diagnostic model development. **(A)**. The differential expression analysis of circulating miR-16, miR-17, miR-27a, miR-19b, and miR-106a in all the 120 BC patients and 50 controls. **(B)**. Higher levels of circulating miR-16, miR-17, miR-27a, miR-19b, and miR-106a in patients at stages II or III or IV than that at stage I. **(C)**, **(D)**. ROC curves showing diagnostic accuracy, AUC and 95% CI of the five models tested in the 120 BC patients and 50 controls. (**E**), **(F)**. Y score distribution plot of the miR-19b-based 3-miRNA model (model 3) in the 120 BC patients **(E)** and 50 normal controls **(F)** showing the true-positive rate (97%) in BC patients and true negative rate (94%) in normal controls. Y score>0.5 was diagnosed as BC.

We combined all the 120 BC patients and 50 normal controls for further validation. Upregulation of circulating miR-16, miR-17, miR-27a, miR-19b, and miR-106a were indicated in BC, compared to control subjects (Figure 5A). Furthermore, all the 5 miRNAs showed higher levels in patients at stages II or III or IV than that at stage I (Figure 5B). ROC analysis indicated the values of AUC, sensitivity and specificity of the 5 diagnostic models (Figures 5C,D). Among the 5 models, miR-19b-based model 3 covering miR-16, miR-19b, and miR-106a showed the highest accuracy of 96% and the highest AUC score of 0.981. Application of the model 3 to the 170 subjects led to 97% of positive rate for BC and 94% of negative rate for normal controls (Figures 5D–F).

**FIGURE 6:**
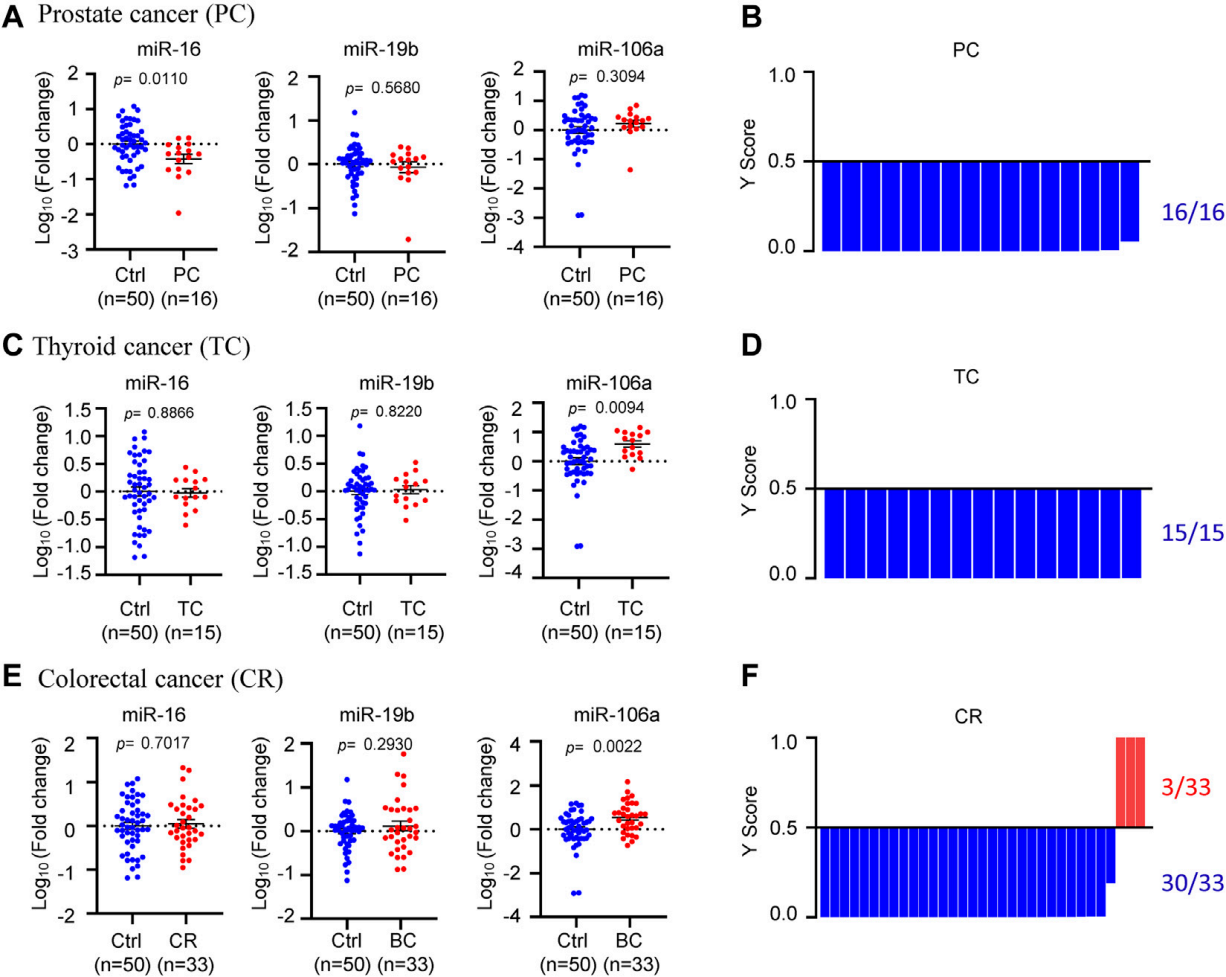
Specificity validation of the model. **(A)**, **(C)**, **(E)**. The expression levels of circulating miR-16, miR-19b, and miR-106a in 16 prostate cancer patients **(A)**, 15 thyroid cancer patients **(C)** and 33 colorectal cancer patients **(E)**, compared to 50 normal controls. **(B)**, **(D)**, **(F)**. Y score distribution plot of the miR-19b-based 3-miRNA model (model 3) in the three other tumor types, showing 100% negative of BC in the 16 prostate cancer patients **(B)**, 100% negative of BC in the 15 thyroid cancer patients **(D)** and 91% negative of BC in the 33 colorectal cancer patients **(F)**. Y score>0.5 was diagnosed as BC.

In order to further validate the specificity of circulating miR-16, miR-19b, and miR-106a in BC, three other tumor types including prostate cancer, thyroid cancer and colorectal cancer were applied for external validation (Figure 6). In the 16 plasma samples from prostate cancer patients, miR-16 showed downregulation while miR-19b and miR-106a did not show significant change, compared with normal controls (Figure 6A). The model 3 diagnosed the 16 samples as 100% negative of BC (Figure 6B). In the 15 plasma samples from thyroid cancer patients, miR-16 and miR-19b were unchanged although miR-106a showed upregulation (Figure 6C). 100% negative of BC was diagnosed by the model 3 (Figure 6D). Similar patterns of the 3 miRNAs were observed in the 33 plasma samples from colorectal cancer patients (Figure 6E), while the model 3 diagnosed 3 of the 33 samples as BC (Figure 6D), which was considered as false positive (9%).

## Discussion

**FIGURE 7:**
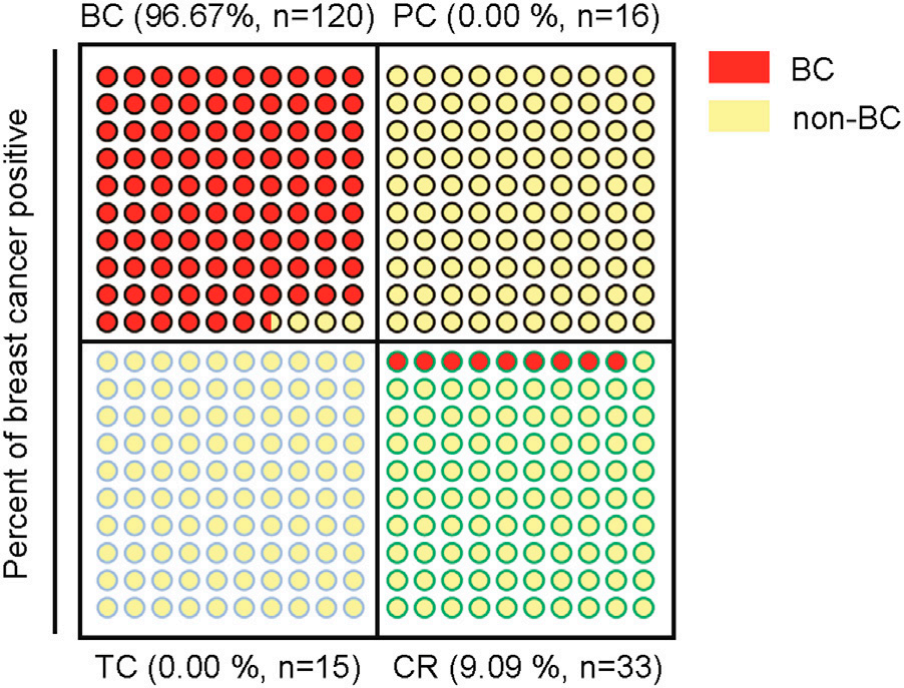
Schematic indication of the specificity for diagnosis of BC of the miR-19b-based 3-miRNA model in 4 different tumor types.

The current study identified 5 circulating miRNAs, miR-16, 19b, 106a, 17, and 27a, as upregulated miRNAs in the plasma of BC, which hold strong potential of diagnosis. Several models were developed using different combinations of these 5 miRNAs. After applying for validation, high sensitivities and high specificities of these models were obtained, especially for the miR-19-based model 3, which was composed of miR-16, 19b and 106a (Figure 7). In consistence with literatures, aberrant expression of these miRNAs has been reported in circulation of patients with breast cancer. For example, the level of miR-16 was higher in serum of invasive intraductal breast carcinoma cases than that in healthy controls (Usmani et al., 2017). More interestingly, 34% of the daughters whose parents or grandparents have had BC showed enhanced expression of miR-16 (Usmani et al., 2017). miR-19b, miR-17 and miR-106a belong to two miRNA paralogs in the human genome, the miR-106a-363 cluster and the miR-17–92 cluster, which showed increased levels in the circulation of breast cancer patients (Hesari et al., 2018; Li et al., 2018). However, there has been no such models combining these miRNAs yet that were developed for diagnosis of BC. Although miR-1,246 and miR-21 in plasma were reported to hold potential to indicate breast cancer (Hannafon et al., 2016), our analysis of miR-1,246 using TCGA database did not show significant change in BC (Supplementary Figure S3). miR-21 showed upregulation in BC patients with a fold change of ∼1.83, but the statistical analysis did not have significance (Figure 2A).

Notably, miR-16 has been frequently used as an internal reference gene for normalization of miRNA analysis either in tissues/cells or in circulation due to its stable expression and high abundance (Wang et al., 2015; Lange et al., 2017). However, here we demonstrated the significantly upregulated levels of miR-16 in the plasma of BC patients, suggesting inappropriateness to set miR-16 as a reference gene in breast cancer. A recent meta-analysis article also verified the high variance in the stability value of miR-16 expression levels in breast cancer (Xiang et al., 2014).

Among the 5 miRNAs we identified in the current study, miR-19b ranked at the top position due to the most significant upregulation in BC and the biggest contribution in the diagnostic model. The model 1 consisted of miR-19b only, which even obtained a high accuracy rate of 92% for BC detection. Furthermore, in the external validation phase using three other tumor types, miR-19b did not show any change in prostate cancer, thyroid cancer and colorectal cancer, further indicating its high specificity in BC. We believe that miR-19b itself only or combination with other genes hold great promise in detection of breast cancer. Nevertheless, further validation using larger patient cohorts with BC and other tumor types will be necessary to strengthen the value of miR-19b and the model 3 before moving forward to the clinical application in diagnosis of breast cancer.

## Data Availability

The original contributions presented in the study are included in the article/Supplementary Material, further inquiries can be directed to the corresponding authors.
