# Supplementary material for: A circulating miR-19b-based model in diagnosis of human breast cancer
Source: Front Mol Biosci. 2022 Sep 16;9:980841. doi: 10.3389/fmolb.2022.980841 (PMC9523242; doi:10.3389/fmolb.2022.980841)

Supplemental Information

Supplemental Table S2: Characteristics of all subjects used in the study.

Supplemental Table S3: Clinical characteristics of breast cancer patients used in the study.

Supplemental Table S4: Home made cancer-related circulating miRNA panel.

Supplemental Figure S1. The expression levels of the 5 miRNA candidates in 764 luminal subtype and 191 basal-like subtype of breast cancer tumors.

Supplemental Figure S2. Formulas and accuracy information of the 5 models developed by binary logistic regression analysis of the miRNA expression data in the independent validation cohort of 80 BC patients and 30 controls.

Supplemental Figure S3. The expression level of the miR-1246 in 1103 breast cancer tumors and 104 normal controls. NS means none significance.

Supplemental Table S2

Table S2 Characteristics of the subjects included in this study

Characteristic	Ctrl	BC	PC	TC	CR
Age (Mean±SD)	55.0±8.1	55.3±12.3	66.5±12.7	44.2±13.4	62.7±14.2
Female/Male	50/0	120/0	0/16	9/6	13/20
Numbers	50	120	16	15	33

BC: breast cancer, PC: prostate cancer, TC: thyroid cancer, CR: colorectal cancer

Supplemental Table S3

Table S3 Clinical characteristics of breast cancer subjects included in this study

Characteristic	Number
Subtype	
Luminal A	8
Luminal B	90
Her2+	20
Basal like	2
Stage	
I	50
II	50
III+IV	20

Supplemental Table S4

Table S4 Home-made cancer-related circulating miRNA panel

	1	2	3	4	5	6	7	8	9	10	11	12
A	let-7a	let-7b	let-7c	let-7d	let-7g	let-7i	H2O	5s	miR-16	H2O	miR-16	miR-17-5p
B	mir-18a	miR-19b	miR-20a	miR-20b	miR-21	miR-23a	miR-29a	miR-29b	miR-29c	miR-30d	miR-34a	miR-34b-3p
C	miR-34c	miR-92a	miR-92b	miR-99b	miR-100	miR-105	miR-106a	miR-122	miR-127-3p	miR-128a	miR-130b	miR-139
D	miR-140-3p	miR-145	miR-151-3p	miR-152	miR-154	miR-155	miR-181a	miR-182	miR-27	miR-192	miR-195	miR-197
E	miR-200a	miR-200b	miR-200c	miR-205	miR-206	miR-221	miR-222	miR-224	miR-320a	miR-320b	miR-320c	miR-320d
F	miR-320e	miR-323a	miR-326	miR-328	miR-329	miR-339-3p	miR-345	miR-346	miR-370	miR-375	miR-409-3p	miR-423-5p
G	miR-424	miR-431	miR-483	miR-484	miR-487b	miR-502-5p	miR-505	miR-584	miR-593	miR-595	miR-638	miR-652
H	miR-654	miR-664a	miR-665	miR-671	miR-675	miR-744	miR-762	miR-766	miR-769	miR-874	miR-877	5s

Supplemental Figure S1

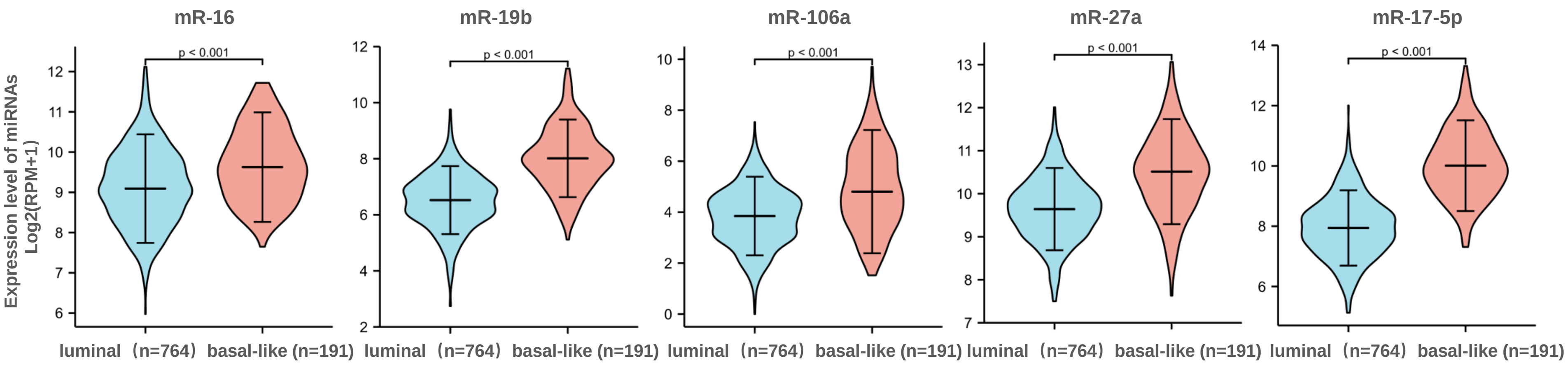

Supplemental Figure S2

Model ID	miRNAs	Model formula (Ctrl: Y<0.5, BC: Y>0.5)	R^2	Accuracy %	AUC	95% CI
Model 1	miR-19b	EXP(-4.379+2350.119*miR-19b)/(1+EXP(-4.379+2350.119*miR-19b))	0.850	94.50% (Ctrl:90.00%, BC:96.30%)	0.989	0.962-1.000
Model 2	miR-16, miR-19b	EXP(-7.261+170.324*miR-16+2901.749*miR-19b)/(1+EXP(-7.261+170.324*miR-16+2901.749*miR-19b))	0.903	96.40% (Ctrl:96.70%, BC:96.30%)	0.993	0.981-1.000
Model 3	miR-16, miR-19b, miR-106a	EXP(-8.382-1682.454*miR-106a+579.027*miR-16+2826.828*miR-19b)/(1+EXP(-8.382-1682.454*miR-106a+579.027*miR-16+2826.828*miR-19b))	0.931	98.20% (Ctrl:96.70%, BC:98.80%)	0.996	0.987-1.000
Model 4	miR-16, miR-19b, miR-27a, miR-106a	EXP(-10.726-10458.587*miR-106a+953.866*miR-16+4405.732*miR-19b+7783.425*miR-27a)/(1+EXP(-10.726-10458.587*miR-106a+953.866*miR-16+4405.732*miR-19b+7783.425*miR-27a))	0.955	98.20% (Ctrl:96.70%, BC:98.80%)	0.998	0.993-1.000
Model 5	miR-16, miR-17, miR-19b, miR-27a, miR-106a	EXP(-12.32-12492.123*miR-106a+931.567*miR-16+4777.146*miR-17-5p+5179.302*miR-19b+10367.007*miR-27a)/(1+EXP(-12.32-12492.123*miR-106a+931.567*miR-16+4777.146*miR-17-5p+5179.302*miR-19b+10367.007*miR-27a))	0.958	98.20% (Ctrl:96.70%, BC:98.80%)	0.999	0.996-1.000

Ctrl: Normal control; BC: Breast cancer

Supplemental Figure S3

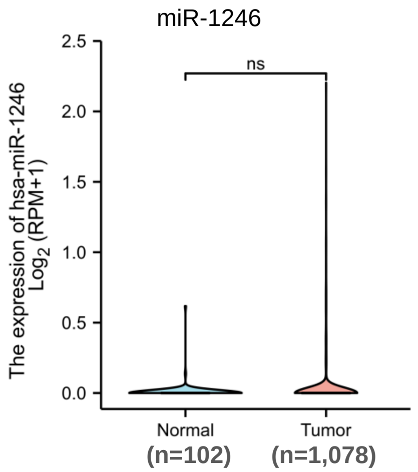

Supplement: Supplementary file 1 [file DataSheet1.pdf]
